# Transcriptome profiling of *Puccinellia tenuiflora* during seed germination under a long-term saline-alkali stress

**DOI:** 10.1186/s12864-019-5860-5

**Published:** 2019-07-17

**Authors:** Xiaoxue Ye, Hao Wang, Xiuling Cao, Xuejiao Jin, Fuqiang Cui, Yuanyuan Bu, Hua Liu, Wenwu Wu, Tetsuo Takano, Shenkui Liu

**Affiliations:** 1 Key Laboratory of Saline-Alkali Vegetation Ecology Restoration in Oil Field (SAVER), Ministry of Education, Alkali Soil Natural Environmental Science Center (ASNESC), Northeast Forestry University, Harbin, 150040 China; 2 State Key Laboratory of Subtropical Silviculture, Zhejiang A&F University, Lin’an, Hangzhou, 311300 China; 3 Asian Natural Environmental Science Center (ANESC), the University of Tokyo, Nishitokyo-shi, Tokyo, 188-0002 Japan

**Keywords:** saline-alkali, *Puccinellia tenuiflora*, transcriptome, noninvasive microtest technique, abiotic stress, mineral elements

## Abstract

**Background:**

*Puccinellia tenuiflora* is the most saline-alkali tolerant plant in the Songnen Plain, one of the three largest soda saline-alkali lands worldwide. Here, we investigated the physicochemical properties of saline-alkali soils from the Songnen Plain and sequenced the transcriptomes of germinated *P. tenuiflora* seedlings under long-term treatment (from seed soaking) with saline-alkali soil extracts.

**Results:**

We found that the soils from Songnen Plain were reasonably rich in salts and alkali; moreover, the soils were severely deficient in nitrogen [N], phosphorus [P], potassium [K] and several other mineral elements. This finding demonstrated that *P. tenuiflora* can survive from not only high saline-alkali stress but also a lack of essential mineral elements. To explore the saline-alkali tolerance mechanism, transcriptional analyses of *P. tenuiflora* plants treated with water extracts from the saline-alkali soils was performed. Interestingly, unigenes involved in the uptake of N, P, K and the micronutrients were found to be significantly upregulated, which indicated the existence of an efficient nutrition-uptake system in *P. tenuiflora*. Compared with *P. tenuiflora*, the rice *Oryza sativa* was hypersensitive to saline-alkali stress. The results obtained using a noninvasive microtest techniques confirmed that the uptake of NO_3_^-^ and NH_4_^+^ and the regulatory flux of Na^+^ and H^+^ were significantly higher in the roots of *P. tenuiflora* than in those of *O. sativa*. In the corresponding physiological experiments, the application of additional nutrition elements significantly eliminated the sensitive symptoms of rice to saline-alkali soil extracts.

**Conclusions:**

Our results imply that the survival of *P. tenuiflora* in saline-alkali soils is due to a combination of at least two regulatory mechanisms and the high nutrient uptake capacity of *P. tenuiflora* plays a pivotal role in its adaptation to those stress. Taken together, our results highlight the role of nutrition uptake in saline-alkali stress tolerance in plants.

**Electronic supplementary material:**

The online version of this article (10.1186/s12864-019-5860-5) contains supplementary material, which is available to authorized users.

## Background

Soil salinization is a major environmental threat to the agriculture industry worldwide and affects approximately 20% of the world’s cultivated land and nearly half of all irrigated land, and the impact is becoming increasingly severe [1]. In fact, it has predicted that 30% and up to 50% of land will be lost due to salinization within the next 25 years and by 2050, respectively [2]. The Songnen Plain, which is located in Northeast China (43°30’–48°40’N, 121°30’–127°00’E), is one of the most important grain-producing areas and serves as an important base for the combination of animal husbandry, agriculture and forestry. However, this area is severely threatened by salinization and alkalization, and a large area of arable land in this plain is being lost. The Songnen Plain is one of the world’s three major soda saline-alkali lands and was formed mainly by the accumulation of sodium bicarbonate (NaHCO_3_) and sodium carbonate (Na_2_CO_3_) [3–6].

The type of salt in a soil is an important factor that determines soil properties. Saline soil is usually formed by the accumulation of neutral salts such as NaCl and Na_2_SO_4_, whereas alkaline soil is formed by the accumulation of alkaline salts such as NaHCO_3_ and Na_2_CO_3_ [7]. Soil salinization can cause oxidative stress, ion toxicity, osmotic stress and metabolic disturbance to plants and thereby affect the growth, division and survival of plant cells [8]. Under salt stress, light utilization and carbon (C) assimilation are inhibited, which leads to an increase in reactive oxygen species (ROS) within chloroplasts and mitochondria and thereby results in oxidative stress [9]. External Na^+^ can negatively impact intracellular K^+^ influx. Alterations in K^+^ ions (due to the impact of high-salinity stress) can disturb the osmotic balance and the functions of stomata and some enzymes. Concentrations of Na^+^ ions greater than 100 mM in soils can be cytotoxic to plants by inhibiting the activity of many important enzymes, cell division and dilatation, membrane disintegration and osmotic imbalance, all of which eventually lead to the inhibition of growth [10]. Some studies have demonstrated that saline-alkali soils are more destructive to plants than saline soils, because plants are able to endure concomitant saline and alkali (high pH) stress [11]. Based on the above-described findings, saline-alkali soils can be more damaging to crops compared with neutral-salt soils because they exhibit oxidative stress, ion toxicity, osmotic stress and high pH, which make it difficult for most crops to grow.

To explore the molecular mechanisms used by plants to survive in the saline-alkali soils in the Songnen Plain, it is necessary to study the halophytes growing in the area. Approximately 74 halophilic seed-producing plants are found in the Songnen Plain [12]. Among these, *Puccinellia tenuiflora* is one of the few species that can survive under severe saline-alkali stress, and in fact, this species is usually used to improve saline-alkali soils [13]. Under experimental conditions, the growth of *P. tenuiflora* is actually stimulated at alkali (NaHCO_3_ and Na_2_CO_3_) levels below 60 mmol/L alkali [14]. The root growth of *P. tenuiflora* was only slightly inhibited by exposure to 150 mmol/L Na_2_CO_3_ for 24 h and 200 mmol/L Na_2_CO_3_ for 12 h [15]. Surprisingly, *P. tenuiflora* can tolerate a maximum of 600 mmol/L NaCl and 150 mmol/L NaHCO_3_ [16]. Under 150 mmol NaCl or 6 mmol NaHCO_3_ treatment, wild-type *Arabidopsis* exhibits both chlorosis and a stunted growth phenotype and ultimately dies [17]. *Glycine soja* seeds can germinate in sodic soils at a pH of 9.02 and cannot survive in nutrient solutions that contain more than 50 mM NaHCO_3_ [18]. Therefore, *P. tenuiflora* is considered a pioneer plant for studying saline-alkali tolerance mechanisms.

Several studies have explored the *P. tenuiflora* molecular features involved in coping with saline-alkali stress. For instance, *P. tenuiflora* seedlings treated with NaCl, Na_2_SO_4_ or both NaHCO_3_ and Na_2_CO_3_ were shown to quench ROS [19], adjust osmotic balance and maintain ion balance under saline-alkali stress [14, 20]. cDNA libraries have been constructed from *P. tenuiflora* to analyze its gene expression profiles under NaHCO_3_ treatment [21, 22], and comparative proteomics has revealed several differentially expressed proteins in *P. tenuiflora* leaves in response to NaCl or Na_2_CO_3_ treatment [15, 23, 24]. Some novel salt tolerance genes in *P. tenuiflora* plants treated with 200 mM NaCl for 0, 6, 12, 24 and 48 h have been discovered [25]. These studies have provided important information for understanding the salt tolerance mechanisms in *P. tenuiflora*. However, most relevant studies focused on single or several salt mixture stresses, which makes it difficult to recapitulate the real situation observed in saline-alkali soils and to analyze the tolerance mechanisms underlying the comprehensive stress responses in natural saline-alkali soils.

In this study, to explore the complex factors that limit plant growth in saline-alkali soils, the physicochemical properties of saline-alkali soils from the Songnen Plain were observed. In addition, transcriptome analyses of *P. tenuiflora* plants germinated in saline-alkali soil extracts and normal soil extracts (control) were performed to reveal the tolerance mechanisms of *P. tenuiflora* adapted to this environment. A noninvasive microtest technique (NMT) was then performed to measure the NO_3_^-^, NH_4_^+^, Na^+^ and H^+^ fluxes in *P. tenuiflora* and *Oryza sativa* roots under saline-alkali stress. Multiple mineral elements were supplied to *O. sativa* by a chelating agent, and the physiological experiment indicated that these supplies enhanced the saline-alkali stress tolerance of rice.

## Results

### Damage from saline-alkali soils to plants and soil physicochemical properties

To explore the growth state under saline-alkali stress conditions, *P. tenuiflora* and the gramineous crop *O. sativa* were selected for saline-alkali tolerance analysis. First, the plants were grown in normal soil to a height of approximately 15 cm, and images of their phenotypes were obtained during this period (Additional file 1: Figure S1 a, c). The seedlings were then watered with saline-alkali soil extracts every three days, and after one week, their phenotype was imaged. The observations revealed that *P. tenuiflora* plants grew normally, with only a few yellow leaves at 7 days after treatment (Additional file 1: Figure S1 b), whereas the leaves of *O. sativa* plants became curled and yellow within 1 day after treatment and eventually died after one week (Additional file 1: Figure S1 d). The survival of the plants was clearly severely restricted after treatment with the saline-alkali soil extracts, and *P. tenuiflora* was more adaptable to saline-alkali stress than was *O. sativa*.

**Fig. 1:**
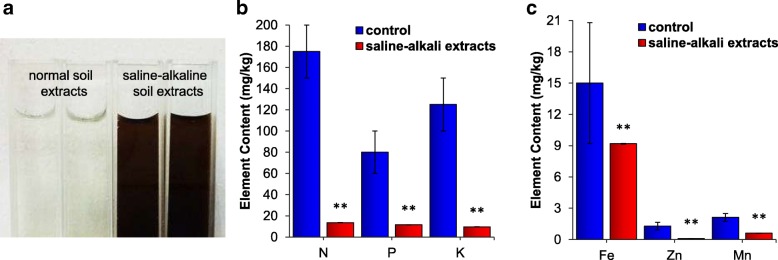
**a** Image of normal soil extracts and saline-alkali soil extracts liquids after centrifugation at 30,000 rpm for 15 min, and passage through a 0.22-μm filter membrane. **b** The contents of essential macronutrients N, P and K in the saline-alkali soil extracts compared with normal soil extracts. **c** The contents of essential micronutrients Fe, Zn and Mn in the saline-alkali soil extracts compared with normal soil extracts. The elements are expressed as milligrams per kilogram

To understand how saline-alkali soils are detrimental to plants growth, liquid extracts of saline-alkali soil from the Songnen Plain were prepared, and the physicochemical properties of the extracts were determined. The soil structure was poor with a high pH of 10.2, and the electrical conductivity was 6,700 μS/cm, which was within the highly conductive range. In addition, the composition of saline-alkali soil extracts is more complex than that of normal soil extracts (Fig. 1a). Moreover, the saline-alkali soil extracts were subjected to overall ion measurements by an inductively coupled plasma-optical emission spectrometer (ICP-OES) and a ThermoFisher ICS-2100 instrument; the contents of 20 major elements are shown in Table 1. Compared with the normal soil extracts [26], the saline-alkali soil extracts were significantly lacking macronutrients, including N, phosphorus (P) and potassium (K) (Fig. 1b), and micronutrients, including Fe, Zn, and manganese (Mn) (Fig. 1c). Because multiple macronutrients and micronutrients are necessary for plant growth, this scarcity might be a limiting factor for plant survival under saline-alkali stress.

**Table 1:** Ion concentrations of saline-alkali soil extracts

Element	mg/L	Element	mg/L
Na	1,909.2	Al	2.63
S	488.1	K	2.5
Ca	44.43	Ti	0.98
F	37.8	Cu	0.88
Cl	12.8	Mo	0.55
Mg	5.79	Ba	0.52
B	5.22	As	0.37
N	4	Mn	0.18
P	3.4	Ni	0.12
Fe	2.75	Zn	0.03

Collectively, the physicochemical property analysis of the saline-alkali soil revealed that the high ion concentration and high pH caused by the presence of NaHCO_3_ salt limited the growth of plants and that the effects of the presence of NaHCO_3_ salt on the soil environment, such as the nutrient elements necessary for plants growth becoming insoluble and changes in the physicochemical properties and structure of the soil, are also important for the inhibition of plant growth in saline-alkali soils.

### The expression of thousands of unigenes was significantly changed under saline-alkali stress

**Fig. 2:**
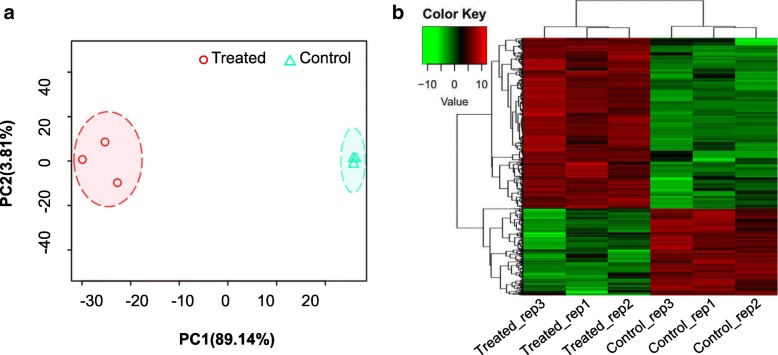
Transcriptome evaluation. **a** PCA of three replicates of the treated and control samples. PC1 explains 89.14% of the variability between the control and treated samples. **b** Heatmap of 3,310 DEGs (2,196 up regulated unigenes and 1,114 down regulated unigenes)

We explored the responses of plants subjected to saline-alkali soil stress, which greatly differ from those of plants subjected to individual salt treatments. To explore the mechanism underlying the notable adaptability of *P. tenuiflora*, transcriptome analyses of three independent biological replicates germinated in normal soil extracts and saline-alkali soil extracts were performed. We obtained ~24 million clean reads from each sample; detailed information on the RNA sequencing (RNA-seq) data is listed in Additional file 9: Table S1. *De novo* transcriptome assembly was performed by using Trinity with clean reads prepared from the control and treated samples, and a total of 42,276 unigenes were retrieved. A principal component analysis (PCA) was conducted to evaluate the reliability of the RNA-seq data (Fig. 2a). PC1 could explain 89.14% of the variability between the control and treated samples, and in contrast to the notable differences between groups, only small differences were detected within each group. These findings together demonstrated that the replicates and groups of the RNA-seq samples show high reliability. Accordingly, we obtained 3,310 differentially expressed unigenes (DEGs: 2,196 upregulated, and 1,114 downregulated) that show significant changes in expression between the control group and the saline-alkali-treated group (Fig. 2b).

To annotate the unigenes assembled by Trinity, a Blastx search against seven databases was performed, and the results are summarized in Additional file 9: Table S3. Among the assembled unigenes, 27,939 (66.08%), 21,372 (50.55%), 16,275 (38.49%), 13,405 (31.71%), 13,040 (30.84%), 11,665 (27.59%) and 16,829 (39.80%) unigenes were successfully annotated with the Nonredundant (Nr), Gene Ontology (GO), Kyoto Encyclopedia of Genes and Genomes (KEGG), Swiss-Prot, Poa, Pfam and Clusters of Orthologous Groups (contains both COG and KOG) databases, respectively. The similarity distribution, E-value distribution and best-hit species distribution were investigated based on the Nr database results (Additional file 2: Figure S2). More details for the GO, KEGG, and COG databases can be found in the Additional file 3: Figure S3, Additional file 4: Figure S4, and Additional file 5: Figure S5, respectively.

**Fig. 3:**
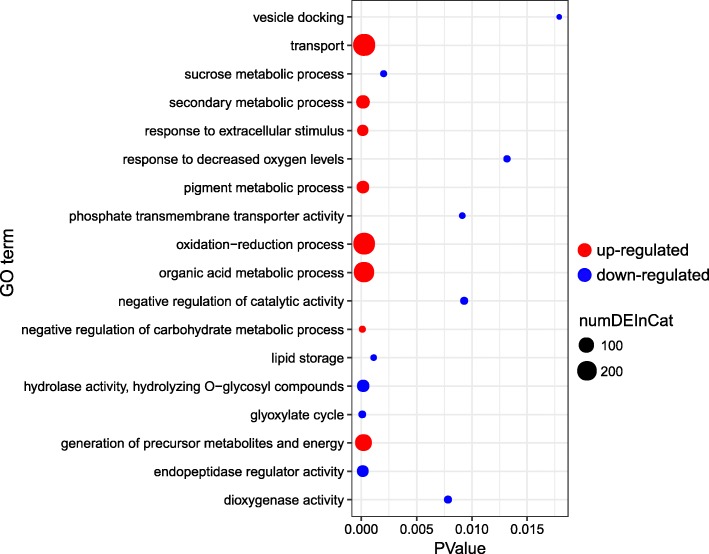
Additional analysis of DEGs. GO term enrichments of the DEGs under saline-alkali conditions

To better understand the function of these DEGs, GO enrichment analysis was conducted using the whole transcriptome dataset as the background. For the biological process group, “transport”, “secondary metabolic process”, “response to extracellular stimulus”, “pigment metabolic process”, “oxidation-reduction process”, “organic acid metabolic process”, “negative regulation of carbohydrate metabolic process” and “generation of precursor metabolites and energy” were the main enriched upregulated subgroups (Fig. 3). Moreover, “vesicle docking”, “sucrose metabolic process”, “response to decreased oxygen levels”, “phosphate transmembrane transporter activity”, “negative regulation of catalytic activity”, “lipid storage”, “hydrolase activity, hydrolyzing O-glycosyl compounds”, “glyoxylate cycle”, “endopeptidase regulator activity” and “dioxygenase activity” were the main enriched downregulated GO terms. Notably, the DEGs involved in the upregulated subgroups were associated with the GO terms “transport”, “oxidation-reduction process” and “organic acid metabolic process”, and these DEGs might function in the saline-alkali tolerance of *P. tenuiflora*. Heat maps based on the expression of these unigenes are shown in Additional file 6: Figure S6, and as shown, these unigenes were not only upregulated but also highly expressed. The organic acid metabolism pathway is shown in Additional file 7: Figure S7, and the red fonts represent upregulated unigenes.

To further validate the reliability of the RNA-seq analysis results, 19 DEGs were randomly selected, and their specific primers were used for real-time quantitative reverse transcription-PCR (RT-qPCR) analysis. The primer information is shown in Additional file 9: Table S2. As shown in Additional file 8: Figure S8 the expression levels measured by RT-qPCR were strongly correlated with the results obtained from the RNA-seq analysis (R^2^ = 0.8073, P = 1.753e-07), demonstrating the reliability of the RNA-seq results.

### DEGs were strongly associated with saline-alkali stress

**Fig. 4:**
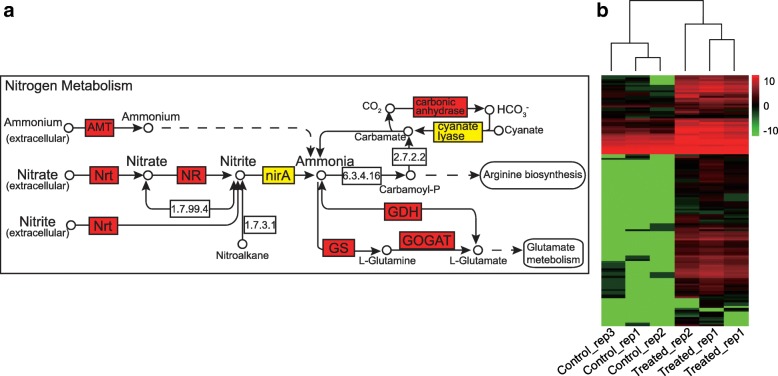
**a** N metabolism pathway under saline-alkali conditions. The expression of unigenes in the Nrt, NR, GS, GDH and GOGAT families is upregulated (red boxes), and members of the nirA and cyanate lyase families are highly expressed (yellow boxes). **b** Heat map of the expression of unigenes involved in the nitrogen (N) metabolism pathway

To further investigate the function of the DEGs, KEGG pathway analysis was performed. Notably, 83 upregulated and highly expressed unigenes were found to belong to the nitrate transporter (Nrt), ammonium transporter (AMT), nitrate reductase (NR), glutamate dehydrogenase (GDH), glutamine synthase (GS), glutamate synthase (GOGAT), carbonic anhydrase, ferredoxin-nitrite reductase (nirA) and cyanate lyase families, all of whose members participate in the nitrogen (N) metabolism pathway (Fig. 4a). N uptake and assimilation are adversely affected by salinity stress. A heat map of the expression of these 83 unigenes is shown in Fig. 4b. The results indicate that these upregulated and highly expressed unigenes involved in N metabolism are modulated to allow the survival of *P. tenuiflora* under the saline-alkali stress and N starvation conditions found in saline-alkali soils.

### The uptake of NO_3_^-^ and NH_4_^+^, the restriction of Na^+^ and the flux of H^+^ were significantly higher in the roots of *P. tenuiflora* than in those of *O. sativa* L. ssp. *japonica*

**Fig. 5:**
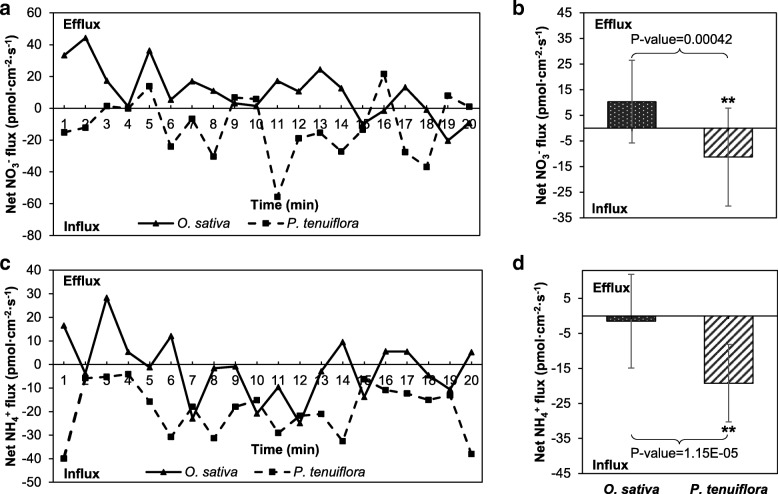
After treatment with saline-alkali soil extracts for 12 h, the roots were transferred to an appropriate measurement solution for continuous flux recording for 10 min. **a** and **c** Real-time net fluxes of NO_3_^-^ and NH_4_^+^ in the roots of *P. tenuiflora* and *O. sativa*. **b** and **d** Mean fluxes of NO_3_^-^ and NH_4_^+^ within the measurement periods. The data were collected every 6 s during the 10 min measurement period. Each point represents the mean of three individual roots, and the bars represent the standard errors of the means. The asterisks show significant differences at *P* < 0.01

Because N metabolism-related unigenes expression in *P. tenuiflora* was markedly induced by saline-alkali stress, the NO_3_^-^ and NH_4_^+^ fluxes in the roots of *O. sativa* and *P. tenuiflora* were measured using an NMT to determine their ability to absorb N under saline-alkali stress. The dynamic changes in the NO_3_^-^ and NH_4_^+^ fluxes are shown in Fig. 5a and c. The results clearly revealed that *P. tenuiflora* maintained an average NO_3_^-^ influx of 11.28 pmol/cm^2^/s, whereas *O. sativa* maintained an average NO_3_^-^ efflux (rather than uptake) of 10.34 pmol/cm^2^/s (Fig. 5b). Similarly, the NH_4_^+^ influxes in *P. tenuiflora* (19.25 pmol/cm^2^/s) were significantly higher than those in *O. sativa* (1.5 pmol/cm^2^/s), despite the presence of NH_4_^+^ influxes in *O. sativa* (Fig. 5d). These results clearly demonstrated that, after exposure to saline-alkali soil extracts, the N uptake rate was notably higher in *P. tenuiflora* than in *O. sativa*, which might be due to the increased expression of N metabolism-related unigenes.

**Fig. 6:**
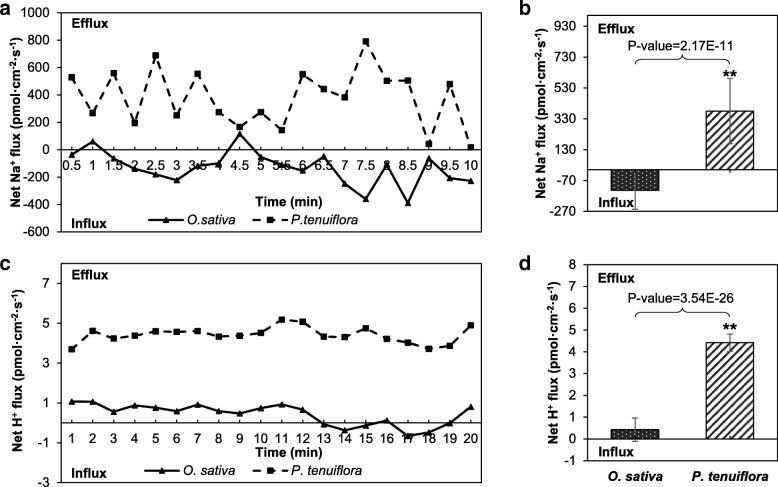
After treatment with saline-alkali soil extracts for 12 h, the roots were transferred to an appropriate measurement solution for continuous flux recording of 10 min. **a** and **c** Real-time net fluxes of Na^+^ and H^+^ in roots of *P. tenuiflora* and *O. sativa*. **b** and **d** Mean fluxes of Na^+^ and H^+^ within the measurement periods. The data were collected every 6 s during the 10 min measurement period. Each point represents the mean of three individual roots, and the bars represent the standard errors of the means. The asterisks show significant differences at P < 0.01

The dynamic changes in Na^+^ and H^+^ fluxes in *P. tenuiflora* and *O. sativa* were also measured in response to saline-alkali stress (Fig. 6a and c). The statistical data revealed that, under saline-alkali stress, *O. sativa* presented an average Na^+^ influx of 132.72 pmol/cm^2^/s (Fig. 6b). This excess Na^+^ would induce severe root growth inhibition or even cell death. In contrast to *O. sativa*, high Na^+^ efflux was observed in *P. tenuiflora*, which allowed this species to maintain low Na^+^ concentrations in its cells and thus withstand the high Na^+^ concentrations found in the saline-alkali soil. In addition, more H^+^ efflux was elicited in *P. tenuiflora* than in *O. sativa* under saline-alkali stress (Fig. 6d); thus, a higher gradient was maintained across the root tissue of *P. tenuiflora* compared with that of *O. sativa*. These reasonable ionic influxes and effluxes might somewhat explain the strong saline-alkali tolerance ability of *P. tenuiflora*.

### Chelating agents and essential elements enhanced the saline-alkali stress tolerance of *O. sativa*

Based on the above-mentioned results, the saline-alkali soil extracts exhibited nutrient deficiencies, and the capacity of *P. tenuiflora* to absorb mineral elements was confirmed by the RNA-seq and NMT results. In consideration of the high pH of the saline-alkali soil extracts, which leads to the formation of insoluble precipitates of metal cations [27, 28], we added microelements via chelating agents. EDTA is a chelating agent with a high affinity constant for the formation of metal-EDTA complexes and is deliberately added to sequester metal ions [28]. Therefore, we evaluated the growth recovery of *O. sativa* under saline-alkali stress by adding Fe(II) EDTA, Zn(II) EDTA, Mn(II) EDTA, KNO_3_, NH_4_NO_3_ and KH_2_PO_4_.

**Fig. 7:**
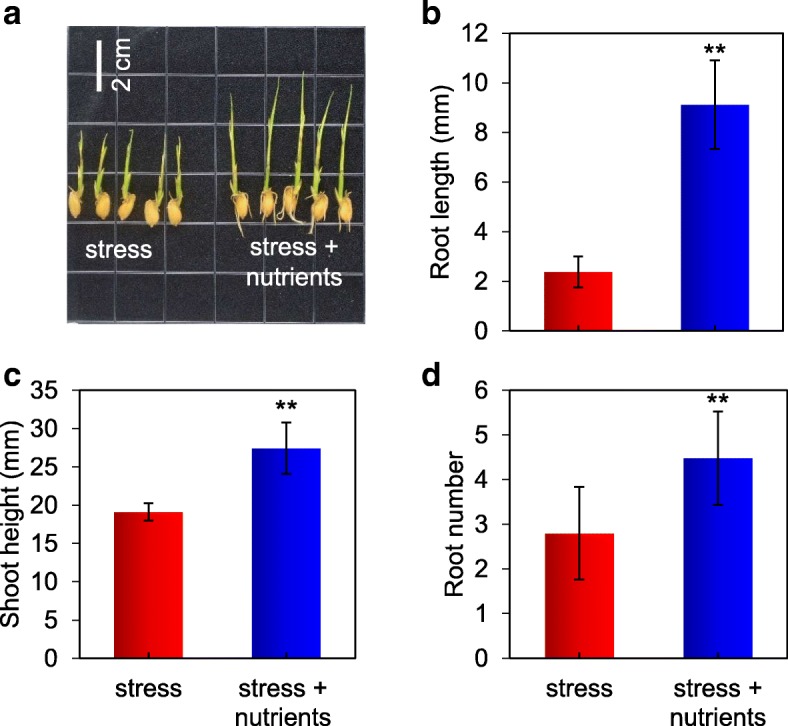
**a** Phenotype of *O. sativa* at the germination stage in the two stress groups; the scale bars represent 2 cm. **b** to **d** Root length, shoot height and root number of the plants in the two stress groups

Two stages of *O. sativa* growth were observed: the germination stage and the seedling stage. At the germination stage, the plants in the mineral element group were clearly larger than those in the stress group (Fig. 7a), and the root length, shoot height and root number were significantly increased in the mineral element group (Fig. 7b-d). The results showed that the root length in the mineral element group was maintained at an average of 9.12 mm, whereas in the stress group, the root length was 2.38 mm. Similarly, the shoot height in the mineral element group (27.44 mm) was significantly higher than that in the stress group (19.12 mm). An average of 4.48 roots was observed in the mineral element group, whereas an average of 2.8 roots was observed in the stress group.

**Fig. 8:**
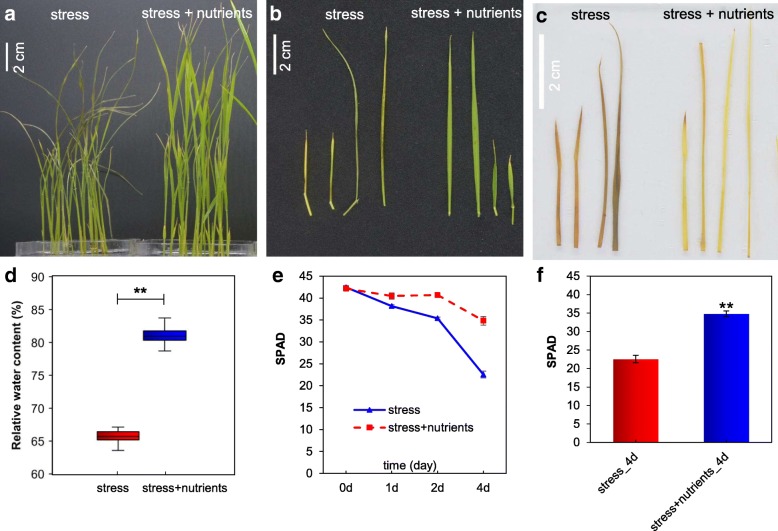
**a** and **b** Phenotype and leaf of *O. sativa* at the seedling stage in the two stress groups; the scale bars represent 2 cm. **c** Comparison of the RWC of *O. sativa* between the two stress groups. **d** DAB staining for H_2_O_2_. (e) to (f) Chlorophyll level measured using a SPAD-502 meter

The phenotype of two-week-old seedlings after 4 days of treatment is shown in Fig. 8a and b. Compared with those in the mineral element group, the leaves of the plants in the stress group were clearly curly and yellow. Although low levels of ROS play roles as signaling molecules in the response to abiotic stress, the generation of massive amounts of ROS in a cell might damage the cell membrane and lead to plant death. Therefore, we used 3,3′-diaminobenzidine (DAB) to estimate the levels of the main ROS, H_2_O_2_, at the seedling stage in the two treatment groups. As shown in Fig. 8c, the plants in the mineral element group had lower levels of H_2_O_2_ than the plants in the saline-alkali soil extract treatment group. We also found that the relative water content (RWC) of the plants in the mineral element group was significantly higher than that of the plants in the saline-alkali soil extract treatment group (Fig. 8d). SPAD units are proportional to the chlorophyll *a* + *b* content, and these values showed that the mineral elements increased the *O. sativa* chlorophyll level compared with that obtained with the extracts (Fig. 8e and f). Together, these results showed that the addition of mineral elements can alleviate salt damage to plants and increase the possibility of their survival.

## Discussion

### Excess ions, high pH, and element deficiency work together to limit plant growth in saline-alkali soils

Saline-alkali soils consist mainly of NaHCO_3_ and Na_2_CO_3_, which cause high levels of pH and Na^+^. Thus, saline-alkali soils can cause oxidative stress, ion toxicity, osmotic stress and metabolic disturbance to plants and thereby affect the survival, growth and division of plant cells. In this study, the pH of the saline-alkali soil extract reached 10.2. Saline-alkali soil is lighter in color than normal soil, but its extract is dark brown. Additionally, the dark brown material remained after passage through a 0.22-μm filter and high-speed centrifugation. We suspect that this color might be due to the presence of soil colloids; thus, the saline-alkali soil is sticky and shows poor permeability. The tiny colloids might also hinder the absorption of water and mineral elements by roots [6]. The content of Na^+^ in the saline-alkali soil extract was 1,909.2 mg/L (Table 1), which is equal to approximately 83 mmol/L, and this concentration could lead to Na toxicity and ion imbalance. Moreover, our ICP results indicated that the saline-alkali soil extracts were lacking various macronutrients and micronutrients (Fig. 1), and this lack, in combination with the high pH and ion toxicity, might restrict plant growth. Collectively, the analyses of the physicochemical properties of the saline-alkali soil extracts revealed the detrimental and complex conditions that plants are exposed to under saline-alkali stress, and elemental deficiency was noticeable among the detected findings.

Plant adaptability under saline-alkali stress has long been investigated. However, very few studies have investigated severe mineral nutrient deficiency, which plays an important role in plant survival in alkaline soils. Under such sophisticated environmental conditions, *P. tenuiflora* exhibits a strong saline-alkali tolerance. To further study the specific metabolic pathways and regulatory mechanisms related to this saline-alkali tolerance, the transcriptomes of *P. tenuiflora* plants treated with saline-alkali soil extracts were analyzed. Although, one transcriptome analysis of *P. tenuiflora* under treatment with 20 mM NaHCO_3_ has been performed, but the low concentration used in the experiment might be not considered as a stress [14, 29]. SO, limited information is available regarding the comprehensive stress responses to excess ions, high pH, and nutrients deficiency, which implies that saline-alkali soil extracts are more representative of natural environmental conditions than single-factor treatments.

### Intracellular ion balance, osmotic adjustment, ROS scavenging and organic acid accumulation are important for the adaption of *P. tenuiflora* to saline-alkali stress

**Fig. 9:**
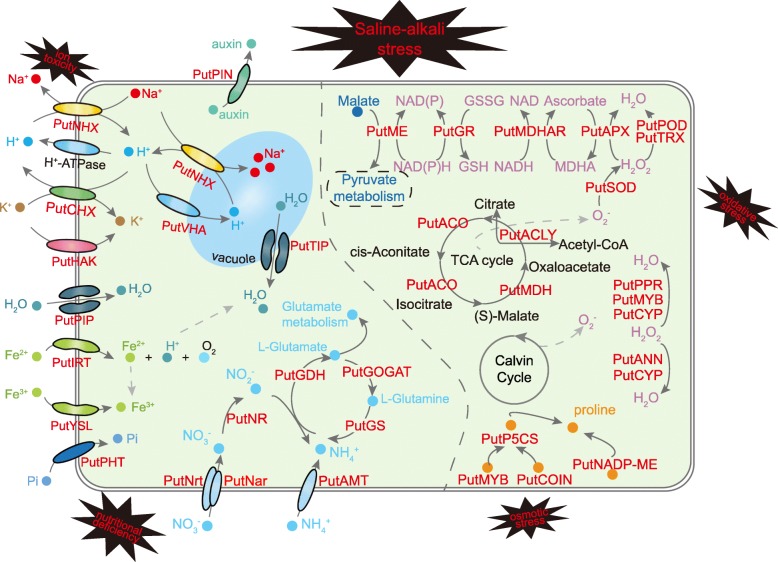
Schematic presentation of systematic salt tolerance mechanism in *P. tenuiflora*

Excess Na^+^ is toxic to cell metabolism, and Na^+^/H^+^ antiporters can transport Na^+^ across membranes by exchanging H^+^ for Na^+^ [30]. Cytoplasmic Na^+^ detoxification involves secreting Na^+^ ions from the cytoplasm to the extracellular space or compartmentalizing them in the vacuole (Fig. 9, Unigenes brief information of Fig. 9 was shown in Additional file 9: Table S4) [19, 31]. The expression level of PutNHX obtained with the saline-alkali soil extracts treatment was significantly higher than that found with the control treatment, which indicates that PutNHX genes not only are important for Na^+^ ion detoxification but also might function in pH regulation under saline-alkali conditions. This result was strongly supported by our NMT results: compared with that in *O. sativa* roots, the influx of Na^+^ in *P. tenuiflora* roots was clearly limited, and H^+^ efflux from *P. tenuiflora* roots in alkali environments was evident (Fig. 6). The results are also in agreement with a previous study, which showed that *P. tenuiflora* leaves exposed to different concentrations of Na_2_CO_3_ could exude salts through their stomata or together with wax [32]. Moreover, intracellular K^+^ and Na^+^ homeostasis is important for the function of many cytosolic enzymes, and the maintenance of a high K^+^ concentration is important for Na tolerance [33]. The CHX gene product has been shown to operate as a K^+^/H^+^ antiporter that controls K^+^ acquisition and homeostasis [34], and the high-affinity K transporter HAK can mediate K^+^ uptake by roots in rice [35]. A previous report also indicated that the maintenance of a high cytosolic K^+^/Na^+^ ratio is important for plant salt tolerance. The upregulation of these unigenes suggested that multiple PutCHX and PutHAK transporters coordinately modulate the cytosolic K^+^/Na^+^ ratio and pH homeostasis of *P. tenuiflora* to withstand saline-alkali stress. The plasma membrane H^+^-ATPase, a proton pump on the plant cell membrane that pumps H^+^ ions to the outside environment to produce a H^+^ transmembrane potential gradient [36], is involved in salt tolerance and is positively regulated by salt stress [37]. The expression levels of a PutPLDA unigene and three V-type proton ATPase unigenes were found to be upregulated in our study, which implies that PutPLDA increases the H^+^-ATPase activity in *P. tenuiflora*. In general, the uptake of some ion, such as phosphate, is driven by the proton gradient generated by plasma membrane H^+^-ATPases [38]. These secondary transport systems of various nutrients and ions, such as those involving PutNHX, PutHAK, PutPHT and PutAMT, need H^+^-ATPases to provide an electrochemical gradient driving force for transmembrane transport (Fig. 9). Consistently, our NMT results revealed a higher H^+^ efflux in *P. tenuiflora* than in *O. sativa* (Fig. 6c and d), which implies that the large proton gradient in *P. tenuiflora* roots regulates the high pH and promotes ion transport.

Proper ion regulation protects cells by enhancing proline accumulation. A previous study revealed that the overexpression of NADP-ME2 could increase the tolerance of plants to salt and osmotic stress [39]. In addition, OsMYB91 and OsCOIN overexpressing plants, which present significantly increased proline levels, exhibit increased tolerance [40, 41] by regulating the expression of OsP5CS, a key enzyme involved in proline biosynthesis [42]. Therefore, the upregulation of the PutNADP-ME, PutMYB, PutCOIN and PutP5CS unigenes observed in this study would mediate the osmotic adaptation of *P. tenuiflora* to saline-alkali soils.

Upon exposure to salt stress, increased ROS levels can act as an important damage-causing agent in plants [1]. Previous studies have revealed that *P. tenuiflora* has evolved multiple antioxidant mechanisms to cope with saline-alkali stress [19]. In the present study, 272 upregulated unigenes were enriched in the oxidation-reduction process (Fig. 3), especially those encoding five critical enzymes involved in ROS scavenging: superoxide dismutase (SOD), L-ascorbate peroxidase (APX), peroxidase (POD), glutathione reductase (GR) and monodehydroascorbate reductase (MDHAR). In addition, OsCYP2 might act as a key regulator that controls the ROS levels by modulating the activities of antioxidant enzymes at the translational level [43], and OsTRXh1 can regulate apoplastic ROS accumulation in rice [44]. OsPPR mutants accumulate more H_2_O_2_ than do wild-type plants [45], and the overexpression of OsANN1 promotes SOD and catalase (CAT) activities, which regulate the H_2_O_2_ contents and redox homeostasis [46]. Under salt stress, OsMYB91 overexpression plants present increased tolerance with a highly enhanced ability to scavenge active oxygen [40]. Thus, upregulated unigenes were enriched in oxidation-reduction process term to reduce the damage of ROS in *P. tenuiflora* under saline-alkali stress.

The accumulation of citric acid in *P. tenuiflora* has been proved to play an important role in pH adjustment [47]. It has been reported that, to maintain an intracellular ionic and osmotic balance under saline or alkaline stress conditions, *P. tenuiflora* can accumulate inorganic anions and organic acids to balance the massive influx of cations [14]. In addition, the formation and accumulation of organic acids play an important role in N metabolism. Previous studies in soybean showed that the accumulation of malic acid through the phloem into the roots will increase the rate of NO_3_^-^ uptake by the roots [48]. It was reported that, due to their high affinity for divalent and trivalent cations, citric acid and other organic acids are able to substitute P from insoluble compounds and thereby make these compounds more soluble and effective for plant uptake [49]. A previous study showed that under conditions of P deficiency, plants can accumulate organic acids in the roots to increase the expression of P transporters [50], which is an important strategy used by plants to adapt to P deficiency. The main form of Fe in alkaline soils is the trivalent Fe compound, which is difficult to dissolve in water; therefore, the amount of organic acids in plants increases to improve the ability of the protoplast membranes of root cells to reduce Fe^3+^, the amount of organic acids in plants increases [51, 52]. As such, understanding the relationship between mineral element uptake and utilization and organic acid metabolism is important for the rational application of fertilizers and genetic engineering to improve the utilization of fertilizer. In our study, organic acid metabolism was consistently one of the most enriched biological process GO terms (Fig. 3). More than two hundred unigenes, including those related to citric acid, malic acid and lactate metabolism, were found to be upregulated, which is similar to the results of previous studies showing that specific organic acids accumulated in alkali-resistant *P. tenuiflora* under salt stress [14, 47]. Our RNA-seq data clearly revealed that the predominant abundance of organic acids increased the ability of *P. tenuiflora* to cope with high pH, balance the influx of cations and improve the uptake of mineral elements (Additional file 7: Figure S7). These unigenes expression trends were consistent with previous results [14, 53], which demonstrate that *P. tenuiflora* might survive in saline-alkali stress by scavenging ROS and accumulating organic acids. Together, the results show that an enhanced ion balance, osmotic adjustment, ROS scavenging and organic acid accumulation coordinately promote the saline-alkali tolerance of *P. tenuiflora* plants under both saline-alkali stress and mineral nutrient deficiency (Fig. 9).

### Enhanced nutrient uptake is an important advantage used by *P. tenuiflora* to adapt to a saline-alkali environment, and the combination of a chelating agent with essential elements can improve the viability of *O. sativa* plants subjected to this stress

**Fig. 10:**
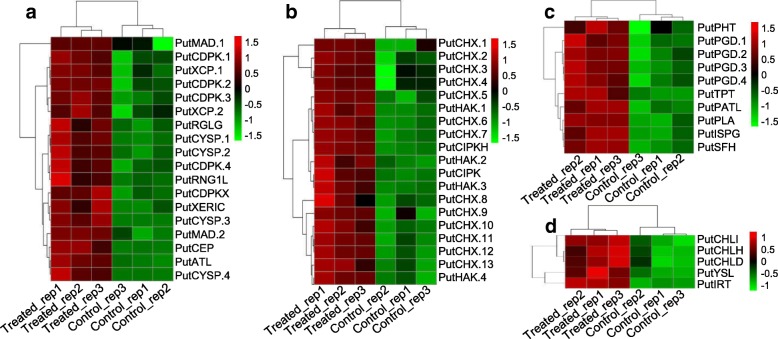
**a** Heatmap of unigenes related to N metabolism. **b** Heatmap of unigenes related to K uptake. **c** Heatmap of unigenes related to P uptake. **d** Heatmap of unigenes related to Fe and Zn uptake

Plant roots require a range of essential mineral elements to survive [54], and N is one of these essential mineral elements due to its important role in the composition of many organic compounds, such as proteins, nucleic acids, chlorophyll, enzymes, vitamins, alkaloids and hormones, which are involved in the transfer of genetic information, organelle formation, photosynthesis, respiration, and nearly all biochemical reactions [55]. The plant uptake of NO_3_^-^ and NH_4_^+^ is an active process mediated by Nrt (together with the high-affinity nitrate transporter-activating protein Nar [56]) and AMT, respectively. N assimilation involves the reduction of NO_3_^-^ to NH_4_^+^, which is ultimately incorporated into amino acids through ammonia assimilation [57]. The N use efficiency (NUE) of chili pepper significantly decreases with increases in salinity conditions [58]. In contrast with previous studies on other plant species, *P. tenuiflora* can maintain higher rates of uptake and assimilation of N, including NO_3_^-^ and NH_4_^+^, compared with rice, as was confirmed by our NMT results. Furthermore, this study identified 83 upregulated and highly expressed unigenes in the N metabolism pathway (Fig. 4). This increased N metabolism might contribute to the N uptake of *P. tenuiflora* under saline-alkali stress combined with N deficiency. Additionally, the NMT was used to measure N uptake, and the results revealed that *P. tenuiflora* exhibited NO_3_^-^ influx whereas *O. sativa* showed NO_3_^-^ efflux (Fig. 5a and b). This difference in N uptake might be due to the expression of unigenes encoding Nrts in *P. tenuiflora*, which could lead to the different saline-alkali tolerances between *P. tenuiflora* and *O. sativa*. Similarly, the uptake of NH_4_^+^ in the roots of *P. tenuiflora* was greater than that in the roots of rice (Fig. 5c and d), which might be due to the upregulated expression of the AMT unigene (Fig. 9). The NMT results were consistent with the RNA-seq results, and both sets of results indicated that under conditions of N deficiency and salinity stress, *P. tenuiflora* exhibits a stronger ability to absorb N from its roots than *O. sativa*. In addition, the upregulation of unigenes related to P, K, Fe and Zn concurrently with that of unigenes related to N demonstrated that *P. tenuiflora* has developed an efficient regulatory mechanism to cope with deficiencies in essential elements, and these upregulated unigenes might have potential utility as a genetic source for engineering saline-alkali tolerance. To survive and maintain meristem activity, the expression levels of MADS-box transcription factor [59], calcium-dependent protein kinase (CDPK [60]), cysteine proteinase (EP3A [61]), and ubiquitin protein ligase (EL5 [62]) unigenes were upregulated in *P. tenuiflora* (Fig. 10a); the products of these unigenes are active under low N conditions. Our results imply that *P. tenuiflora* has its own set of mechanisms to ensure vigorous vitality under conditions of N deficiency. In addition, substantial changes in the expression of some unigenes in response to various mineral element deficiencies were also observed in *P. tenuiflora* after exposure to saline-alkali soil extracts. With respect to K deficiency, expression of the K transporter PutHAK [63], cation/H^+^ antiporter PutCHX [64], and CBL-interacting protein kinase PutCIPK [65], which participate in the competition with Na^+^ during K starvation and salt stress, was upregulated significantly (Fig. 10b). The upregulation of these unigenes is likely critical for modulating K transport. Furthermore, the mineral nutrient P is also of particular importance due to the complex array of metabolic processes in which it is involved. PutPHT is a high-affinity transporter for external inorganic phosphate whose function is similar to that of PHT1-4 in *Arabidopsis thaliana*, which acts as a phosphate symporter under both low- and high-Pi conditions [66]. The unigenes for 6-phosphogluconate dehydrogenase, a triose phosphate/phosphate translocator, patellin-5, phospholipase A1-II, 4-hydroxy-3-methylbut-2-en-1-yl diphosphate synthase and phosphatidylinositol/phosphatidylcholine transfer protein SFH (abbreviated PutPGD, PutTPT, PutPATL, PutPLA, PutSPG and PutSFH, respectively), which encode products that also participate in processes related to P metabolism, were annotated based on their similarity with the Swiss-Prot database, as shown in Fig. 10c. These upregulated unigenes might function in a coordinated manner to cope with P deficiency in saline-alkali soils. Similarly, the expression of magnesium (Mg)-chelatase unigenes was also induced (Fig. 10d), which was in agreement with previous findings showing that the PutCHLH, PutCHLI and PutCHLD unigenes are required for the effective uptake of Fe under conditions of Fe deficiency [67]. Expression of the Fe(2^+^) transporter PutIRT [68] and the Fe(3^+^) metal-nicotianamine transporter PutYSL [69] unigenes, which were considered to regulate Fe and Zn uptake for *P. tenuiflora* growth, was also induced. The upregulation of all these unigenes suggests that *P. tenuiflora* has evolved a comprehensive regulatory mechanism to cope with a lack of essential elements and saline-alkali stress.

A previous study revealed that dietary deficiencies of essential micronutrients such as Zn and Fe affect more than two billion people worldwide [70]. The diets of more than two-thirds of the global population lack one or more essential mineral elements that can be remedied by increasing the concentrations and/or bioavailability of mineral elements in produce (biofortification) [71]. During the past 3 decades, the relationship between maize grain yield (GY) formation and N uptake dynamics has been increasingly acknowledged in the scientific literature [72]. K transport in general and K channels in particular represent important targets and are mediators of cellular responses during different developmental stages in a plant’s life cycle [73]. Moreover, in saline-alkali soils, carbonate, bicarbonate and high pH lead to the formation of insoluble precipitates of metal cations [27, 28], which leads to the absence of essential microelements. EDTA is a powerful complexing agent of metals and is a highly stable molecule. Synthetic chelating ligands form Fe-ligand (Fe–L) complexes in the rhizosphere, and these complexes increase the solubility of Fe and therefore its bioavailability and uptake by plants [74]. Moreover, a previous study showed that EDTA has no effect on the pH of the soil solution [75]. Accordingly, we added microelements via chelating agents. Due the low amounts of essential elements in the saline-alkali extracts and the upregulation of nutrient-related unigenes in resistant *P. tenuiflora* during saline-alkali stress, the nontolerant rice cultivar Nipponbare was used to explore whether supplementation with essential elements could result in plant survival. The assessment of this rice cultivar at two periods and the measurement of multiple indicators verified the important roles of mineral elements in saline-alkali tolerance. Thus, supplementation with mineral elements and improvement in the uptake of mineral elements represent important and potential strategies for strengthen the tolerance of crops to saline-alkali stress.

## Conclusions

Our study revealed that plants in saline-alkali soils suffer from multiple constraints. In addition to conditions of excess ion toxicity, osmotic stress, oxidative stress and high pH, deficiencies in soluble mineral elements are also a nonnegligible limiting factor. The RNA-seq and NMT results revealed the regulatory mechanism used by *P. tenuiflora* under saline-alkali stress; this mechanism involved maintenance of the intracellular ion balance, osmotic adjustment, ROS scavenging, and organic acid accumulation, particularly the uptake and assimilation of essential mineral elements (Fig. 9). On the other hand, measurements of the root length, shoot height, root number, ROS accumulation, RWC and chlorophyll content of rice at both the germination and seedling stages showed that supplementation with a chelating agent combined with essential elements can help rice survive under saline-alkali stress. These findings provide new insights into the saline-alkali tolerance mechanism of plants and the effective utilization of saline-alkali land resources in the future.

## Methods

### Source of soils and seeds

Soils were collected from the Alkali Soil Natural Environment Science Center of Northeast Forestry University, Anda practice base, Heilongjiang Province, Northeast China. The saline-alkali soil used for extraction was collected from the wild “barren” lands in the Songnen Plain at a depth of 0–10 cm depth. The normal soils used in the experiment were obtained from croplands in the Songnen Plain at 0–10 cm depth. Wild-type of *P. tenuiflora* seeds were provided by the Alkali Soil Natural Environment Science Center of Northeast Forestry University, Harbin, China (materials posted when needed). O. sativa L. ssp. japonica seeds were provided by the Asian Natural Environmental Science Center (ANESC) of the University of Tokyo, Japan. No specific permissions were required for these activities because sampling process did not affect any endangered or protected species.

### Preparation of saline-alkali soil extracts

The uniform soil samples were passed through a sieve and then mixed separately with deionized water at a volume ratio of 1:4. The soils were thoroughly mixed, which was essential, and then incubated overnight. The resulting liquid, which represented the crude extract, was then centrifuged for 15 min and filtered through a 0.22-μm microporous membrane. Both types of soil extracts were prepared successfully (Fig. 1a).

### Physicochemical properties of saline-alkali soils

#### Determination of pH values and electrical conductivity

The pH values of the saline-alkali soil extracts were measured using a Sartorius PB-10 instrument, which has a range of 0 to 14.00 and a resolution of ±0.01. We measured the electrical conductivity of the soil extracts using a DDS-307 electrical conductivity meter (Shanghai Leici), which has a measurement range of 0.00 μS/cm to 100 mS/cm, a basic error of ±1.0% full scale (FS), and a stability of ±0.33% FS/3 h.

### Measurement of ion concentrations in the saline-alkali soil extracts

The cation and anion concentrations in the saline-alkali soil extracts were determined using a Perkin Elmer Optima 8300 ICP-OES instrument and a ThermoFisher ICS-2100 instrument, respectively. The saline-alkali soil extracts were diluted 10-fold for detection, and the measurements were then converted to the original concentration. The analytes detected included Na, S, calcium (Ca), fluorine (F), chlorine (Cl), Mg, boron (B), N, P, Fe, aluminum (Al), K, titanium (Ti), copper (Cu), molybdenum (Mo), barium (Ba), arsenic (As), Mn, nickel (Ni) and Zn. The instrument measured the intensity of light emitted by the thermally excited elements at characteristic wavelengths related to the elemental concentrations.

### Plant material, growth conditions and stress treatments

The field-collected seeds were surface-sterilized with a 75% ethanol solution and then sequentially rinsed three times with sterilized distilled water, once with a 0.1% NaClO solution and three times with sterilized distilled water. The seeds were then sown in normal soil extract (control group) and saline-alkali soil extract (treated group) separately for germination. The greenhouse was maintained at 28 °C (day) and 22 °C (night) under a 12/12 h photoperiod. The 3-mm-high aboveground parts of *P. tenuiflora* seedlings (three replications per group) to be used for RNA extraction were rapidly washed, frozen in liquid N_2_ and stored at -80 °C separately.

### Transcriptome sequencing, *de novo* assembly and functional annotation

Approximately 8 μg of RNA was isolated using the TRIzol method. The quality and quantity of the RNA samples were assessed using a NanoDrop instrument and an Agilent 2100 Bioanalyzer. High-quality RNA with an RNA integrity number (RIN) > 8 and of sufficient quantity was used for construction of the sequencing library. The cDNA libraries were sequenced on an Illumina HiSeq 2000 platform to generate 150 bp paired-end reads. Trimmomatic [76] was used to remove the Illumina adapter contamination and for trimming the reads and clipping the low-quality bases, and at least 8 Gb of clean reads were obtained. Kraken software [77] was further used to delete the reads from bacteria. The clean reads were subsequently *de novo* assembled using Trinity software [78], and the constructed “genes” were referred to as unigenes in this study.

The unigene sequences were aligned to the Nr, Swiss-Prot, COG and Pfam databases using Blastx with an E-value of 1e-5. To obtain a high confidence of gene annotations, we constructed a database named Poa, which comprised all currently annotated Gramineae plant proteins. GO annotation was obtained using the R package GOseq [79], and visualization was performed by WEGO [80] software. The unigene sequences were also aligned to the KEGG [81] pathway database using the KEGG Automatic Annotation Server (KAAS) [82]. RNAMMER [83] was used for rRNA prediction, and unigenes marked as rRNA were removed.

### Identification of DEGs

The unigene expression levels of both the control group and the treatment group were calculated. An analysis of the DEGs between the two groups was performed using the R packages DEseq2 [84], edgeR [85] and voom [86]. We defined DEGs as those that had a false discovery rate (FDR) ≤ 0.01 and an absolute value of log2-fold change ≥ 1 obtained from at least two of the above-mentioned packages. GO enrichment analysis of the DEGs was performed using ClueGO [87].

### RT-qPCR analysis

RT-qPCR was performed to validate the gene expression results obtained from the RNA-seq analysis. First, cDNA was reverse-transcribed from 1 μg of total RNA for each sample using a High-Capacity cDNA Reverse Transcription Kit. The gene-specific primers used in this analysis (Additional file 9: Table S2) were designed using Primer-Blast (NCBI) software [88]; the PCR product length was maintained between 150 bp and 300 bp, and the primer purification method involved iPAG. The *P. tenuiflora* tubulin gene was used as an internal reference in the RT-qPCR analysis. The 20 μl PCR mixture used was composed of 1 μl of cDNA, 0.8 μl of the specific primers, 10 μl of SYBR Premix DimerEraser, and 8.2 μl of ddH_2_O. All the reactions were performed using default parameters, and the specificity of the reactions was verified through melting curve analysis. The real-time PCR analysis incorporated the results of three biological and three technical replicates.

### Analysis of the ion fluxes in *P. tenuiflora* and *O. sativa* roots using the NMT

The net NO_3_^-^, NH_4_^+^, Na^+^ and H^+^ fluxes within *P. tenuiflora* and *O. sativa* root meristematic regions were measured at the YoungerUSA Xuyue (Beijing) BioFunction Institute by using NMTs (NMT100 Series, YoungerUSA, LLC, Amherst, MA 01002, USA; Xuyue (Beijing) Sci. & Tech. Co., Ltd., Beijing, China) and imFluxes V2.0 (YoungerUSA, LLC, Amherst, MA 01002, USA) software. The NMT is capable of noninvasively measuring the real-time flux of various ions by simultaneously integrating and coordinating different voltage signal collections, motion control, and image capture.

To observe the ion influx or efflux within *P. tenuiflora* and *O. sativa* roots, the plants were treated with saline-alkali soil extracts for 12 h. Net NO_3_^-^, NH_4_^+^, Na^+^ and H^+^ fluxes in the meristematic zone of the roots were monitored, and a steady flux was recorded for 20 min. The test solution for NO_3_^-^ consisted of 0.1 mM CaCl_2_, 0.1 mM KNO_3_ and 0.3 mM 2-(N-morpholino) ethanesulfonic acid (MES), and the test solution for NH_4_^+^ consisted of 0.1 mM CaCl, 0.1 mM NH_4_Cl and 0.3 mM MES. The test solution for Na^+^ consisted of 0.1 mM CaCl, 0.1 mM KCl, 0.5 mM NaCl and 0.3 mM MES, and the test solution for H^+^ consisted of 0.1 mM CaCl, 0.1 mM KCl and 0.3 mM MES. The pH of each test solution was 6, and each experiment was repeated four times. The differences in microvolts (△μV) were then exported as raw data before they were imported and converted into the net Na^+^ (the same as H^+^, NO_3_^-^, NH_4_^+^) fluxes by using JCal V3.3 (a free Microsoft Excel spreadsheet; youngerusa.com or xbi.org) [89].

### Supply of chelating agents combined with essential elements to *O. sativa* under saline-alkali conditions

The seeds used to investigate the germination stage were sterilized with 0.1% NaClO for 30 min and then washed with distilled water. The seeds were then soaked in distilled water for 12 h and pregerminated in Petri dishes that contained three layers of moistened filter paper for 24 h. Upon emergence, ~30 seeds were separately placed in saline-alkali soil extracts that contained or did not contain essential elements (Fe(II) EDTA, Zn(II) EDTA, Mn(II) EDTA, KNO_3_, NH_4_NO_3_, and KH_2_PO_4_) for germination. The components of the elements in the nutrient solution are shown in Table 2. To control the consistency of the treatment conditions, the pH of the solution was adjusted to 9.5, and 1 ml was dissolved in 100 ml of soil extracts. Considering the poor resistance of *O. sativa*, we diluted the soil extract once. The shoot height, root length and root number of 20 randomly selected seedlings were manually measured on day 10.

**Table 2:** Elements Composition of the multi nutrient solution

Component	Relative molecular mass	Concentration (mmol/L)
KNO_3_	101.1	469.83
NH_4_NO_3_	80.04	515.37
KH_2_PO_4_	136.1	31.23
EDTA - Mn	389.1	2.49
EDTA - Fe	367.2	2.50
EDTA - Zn	471.6	0.75

The seeds used to investigate the seedling stage were washed and pregerminated using the same protocol used in the germination investigation. The *O. sativa* plants were grown in 1/8-strength Murashige and Skoog (MS) solution, which was renewed every 3 days. Two-week-old seedlings were transferred to saline-alkali soil extracts that contained or did not contain essential elements, and the seedlings were then allowed to grow for 72 h. The RWC was recorded by weighing the individual seedlings. DAB staining was also performed for measurement of the H_2_O_2_ level according to previously described methods [90]. The SPAD-502 meter (Konica Minolta Sensing, Osaka, Japan) is a hand-held device that is widely used for rapid, accurate and nondestructive measurements of leaf chlorophyll concentrations [91]. Ten independent SPAD values were recorded per treatment using different plants.

## Additional files

Additional file 1: **Figure S1.** (a) and (b) Phenotypes of *P. tenuiflora* and *O. sativa* seedlings in normal soil. (c) and (d) Seedlings watered with saline-alkali soil extract for one week. (e) and (f) Scanning electron microscope micrographs of saline-alkali soil extracts. (TIF 12908 kb)

Additional file 2: **Figure S2.** BLAST results from the Nr database. (a) Similarity distribution. (b) Best-hit species classification. (c) E-value distribution. (PDF 120 kb)

Additional file 3: **Figure S3.** GO classifications of assembled unigenes from *P. tenuiflora* obtained using Blast2GO. The unigenes were annotated in three main categories: cellular component, molecular function and biological process. The x-axis indicates the subcategories, and the y-axis indicates the number of unigenes. (PDF 273 kb)

Additional file 4: **Figure S4.** KEGG pathways of assembled unigenes from *P. tenuiflora*. The x-axis indicates the categories, and the y-axis indicates the number of unigenes. (PDF 189 kb)

Additional file 5: **Figure S5.** COG classifications of assembled unigenes from *P. tenuiflora*. Of the 42,276 *de novo*-assembled unigenes, 16,829 were annotated and separated into 23 categories. (PDF 141 kb)

Additional file 6: **Figure S6.** Additional analysis of DEGs. (a) Heatmap of 278 transport unigenes that share a significantly upregulated pattern. (b) Heatmap of 272 oxidation-reduction process unigenes that share a significantly upregulated pattern. (c) Heatmap of 271 organic acid metabolic process unigenes that share a significantly upregulated pattern. (PDF 162 kb)

Additional file 7: **Figure S7.** Organic acid metabolism under saline-alkali conditions; the red font indicates the upregulated unigenes. (PDF 107 kb)

Additional file 8: **Figure S8.** Correlation analysis of 19 randomly selected DEGs based on the qRT-PCR and RNA-seq data. Pearson correlation coefficients are shown (R^2^ = 0.8073) (*P* < 0.001). (PDF 78 kb)

Additional file 9: **Table S1.** Summary of Illumina transcriptome sequencing for *P. tenuiflora.* **Table S2.** Primers information. **Table S3.** Functional annotation of the unigenes of *P. tenuiflora.* **Table S4.** Unigenes in salt tolerance mechanisms schematic in *P. tenuiflora*. (XLSX 13 kb)

## Data Availability

All the sequencing data generated in this study have been deposited in the Sequence Read Archive (SRA) database under accession number PRJNA491308.

## Abbreviations

AMT: ammonium transporter
CDPK: calcium-dependent protein kinase
COG: Clusters of Orthologous Groups
DAB: 3,3’-diaminobenzidine
DEGs: differentially expressed genes
EDS: energy dispersive spectroscopy
EL5: ubiquitin protein ligase
EP3A: cysteine proteinase
GDH: glutamate dehydrogenase
GO: Gene Ontology
GOGAT: glutamate synthase
GS: glutamine synthase
ICP-OES: inductively coupled plasma-optical emission spectrometer
KEGG: Kyoto Encyclopedia of Genes and Genomes
Nar: high-affinity nitrate transporter-activating protein
nirA: ferredoxin-nitrite reductase
NMT: noninvasive microtest techniques
NR: nitrate reductase
Nr: nonredundant
Nrt: nitrate transporter
*P. tenuiflora*: *Puccinellia tenuiflora*
PCA: principal component analysis
ROS: reactive oxygen species
RT-qPCR: real-time quantitative reverse transcription-PCR
RWC: relative water content
