# Supplementary figures and images for: Transcriptome profiling of Puccinellia tenuiflora during seed germination under a long-term saline-alkali stress
Source: BMC Genomics. 2019 Jul 17;20:589. doi: 10.1186/s12864-019-5860-5 (PMC6637651; doi:10.1186/s12864-019-5860-5)

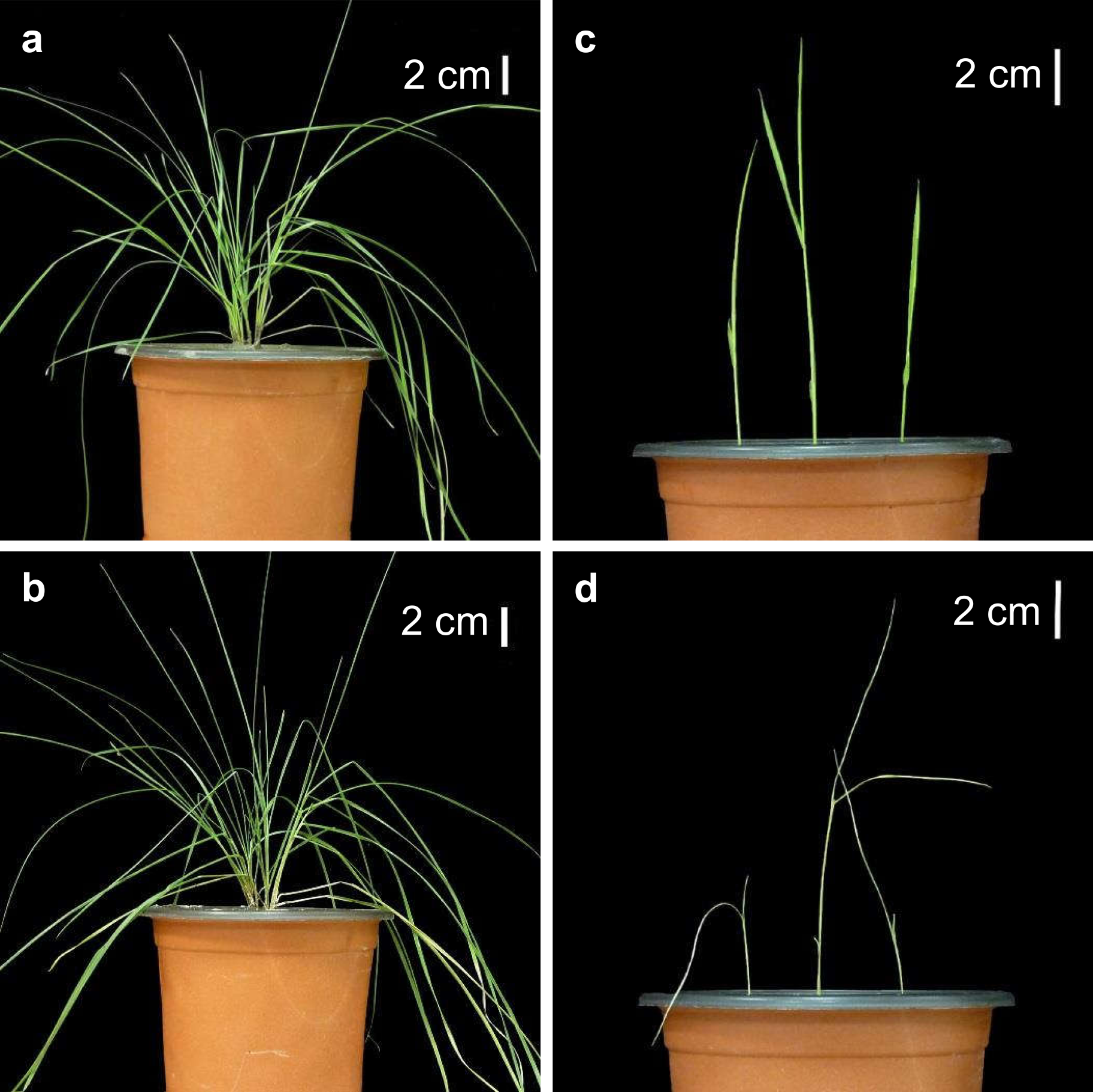

Supplement: Supplementary file 1 — Figure S1. (a) and (b) Phenotypes of P. tenuiflora and O. sativa seedlings in normal soil. (c) and (d) Seedlings watered with saline-alkali soil extract for one week. (e) and (f) Scanning electron microscope micrographs of saline-alkali soil extracts. (TIF 12908 kb) [file 12864_2019_5860_MOESM1_ESM.tif]

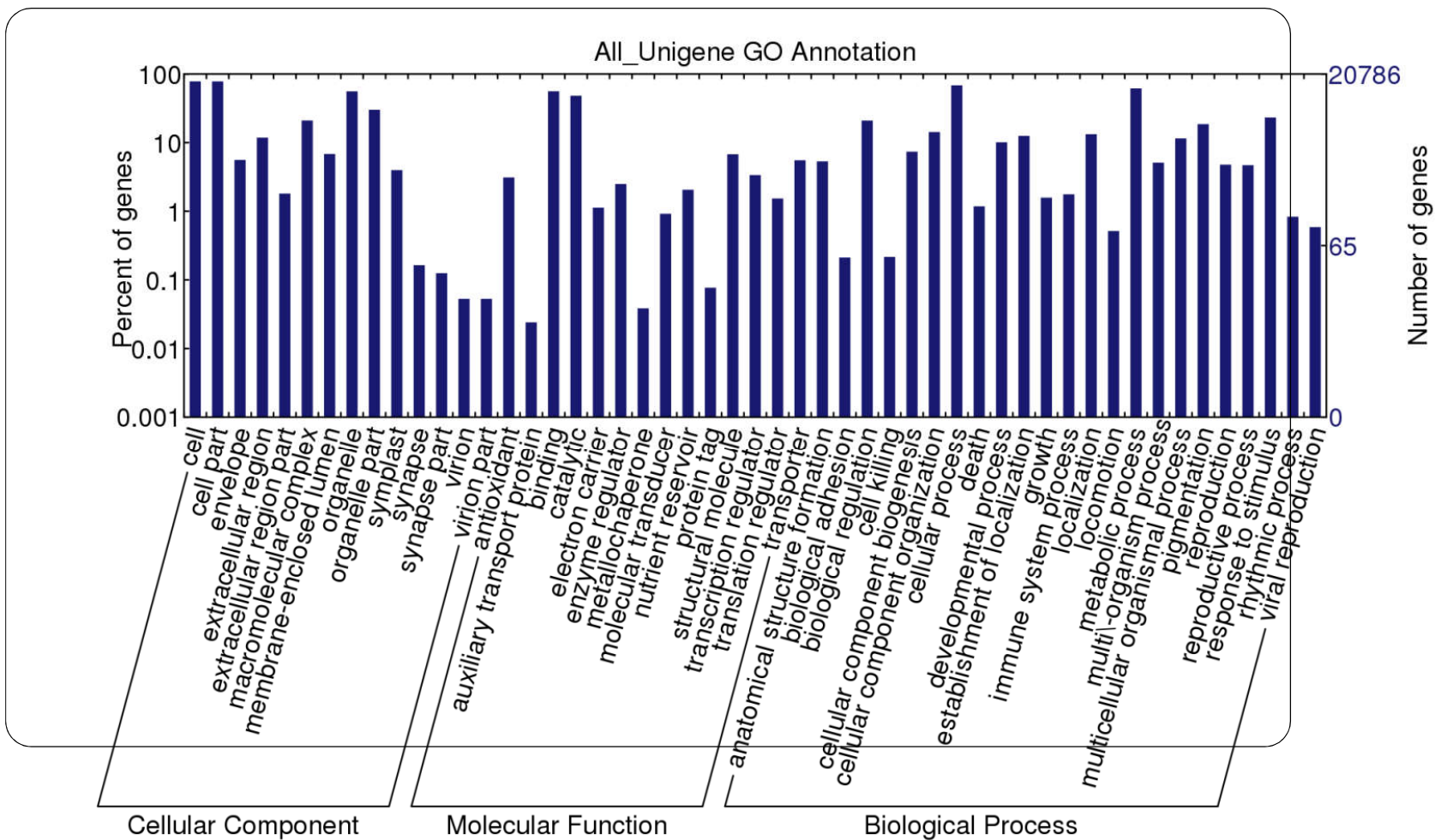

Supplement: Supplementary file 3 — Figure S3. GO classifications of assembled unigenes from P. tenuiflora obtained using Blast2GO. The unigenes were annotated in three main categories: cellular component, molecular function and biological process. The x-axis indicates the subcategories, and the y-axis indicates the number of unigenes. (PDF 273 kb) [file 12864_2019_5860_MOESM3_ESM.pdf]

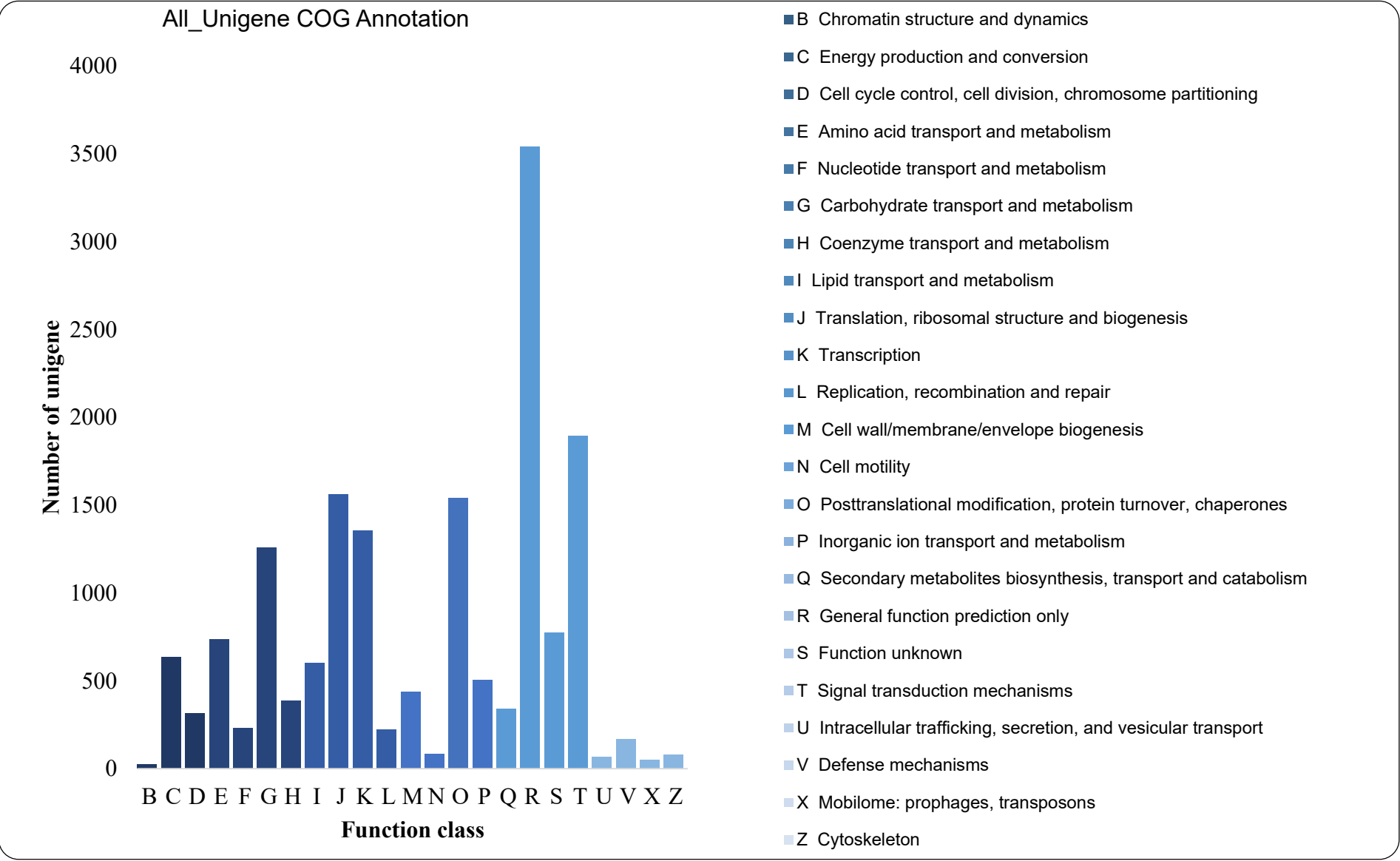

Supplement: Supplementary file 5 — Figure S5. COG classifications of assembled unigenes from P. tenuiflora. Of the 42,276 de novo-assembled unigenes, 16,829 were annotated and separated into 23 categories. (PDF 141 kb) [file 12864_2019_5860_MOESM5_ESM.pdf]

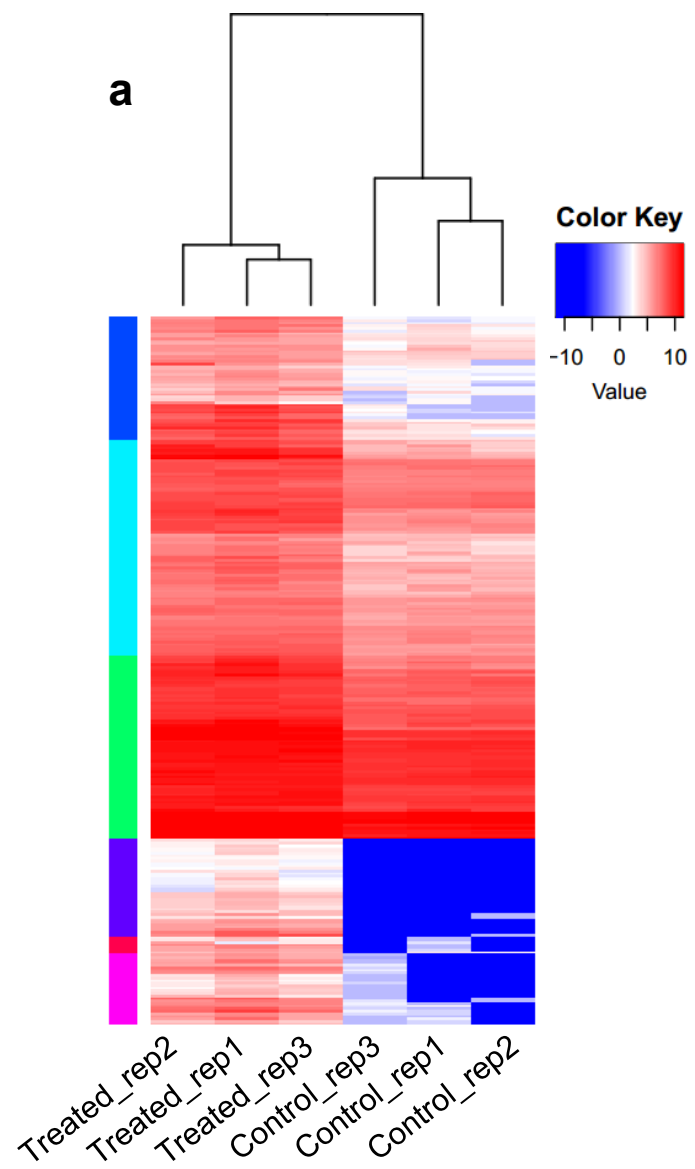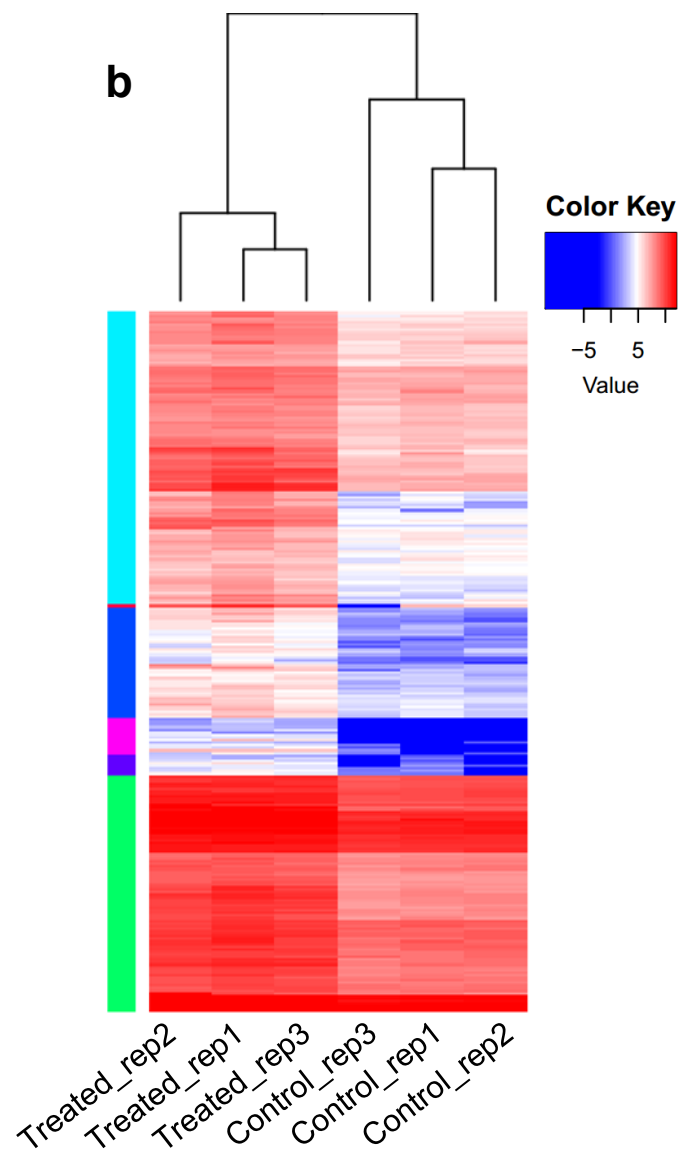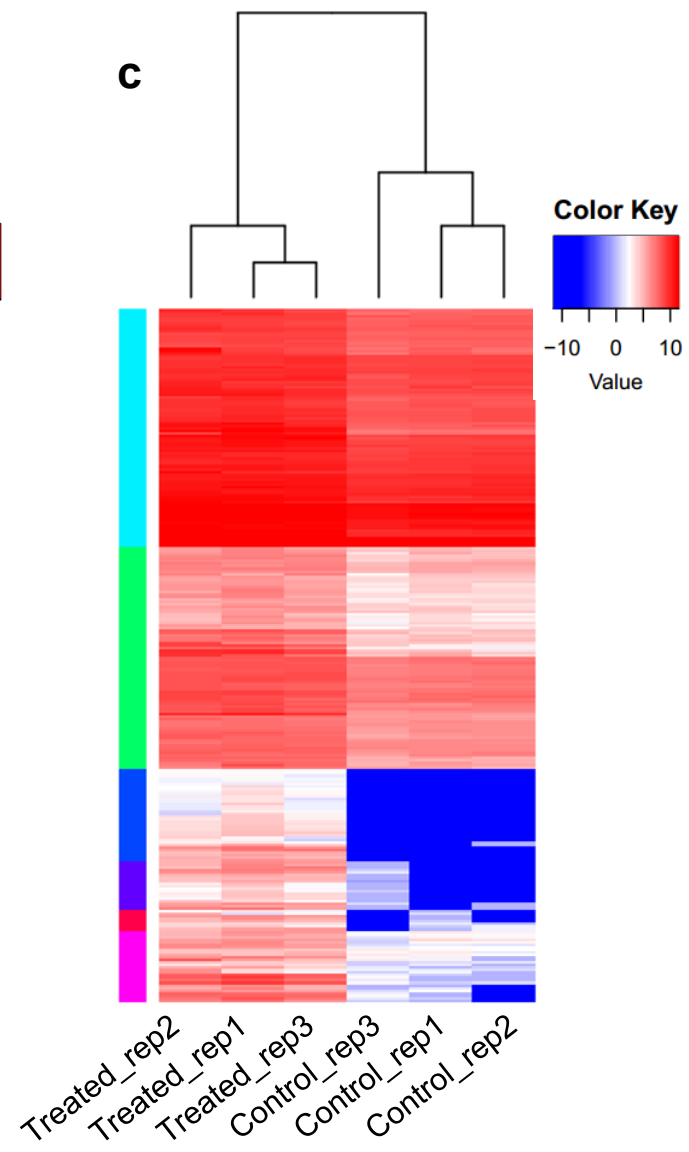

Supplement: Supplementary file 6 — Figure S6. Additional analysis of DEGs. (a) Heatmap of 278 transport unigenes that share a significantly upregulated pattern. (b) Heatmap of 272 oxidation-reduction process unigenes that share a significantly upregulated pattern. (c) Heatmap of 271 organic acid metabolic process unigenes that share a significantly upregulated pattern. (PDF 162 kb) [file 12864_2019_5860_MOESM6_ESM.pdf]

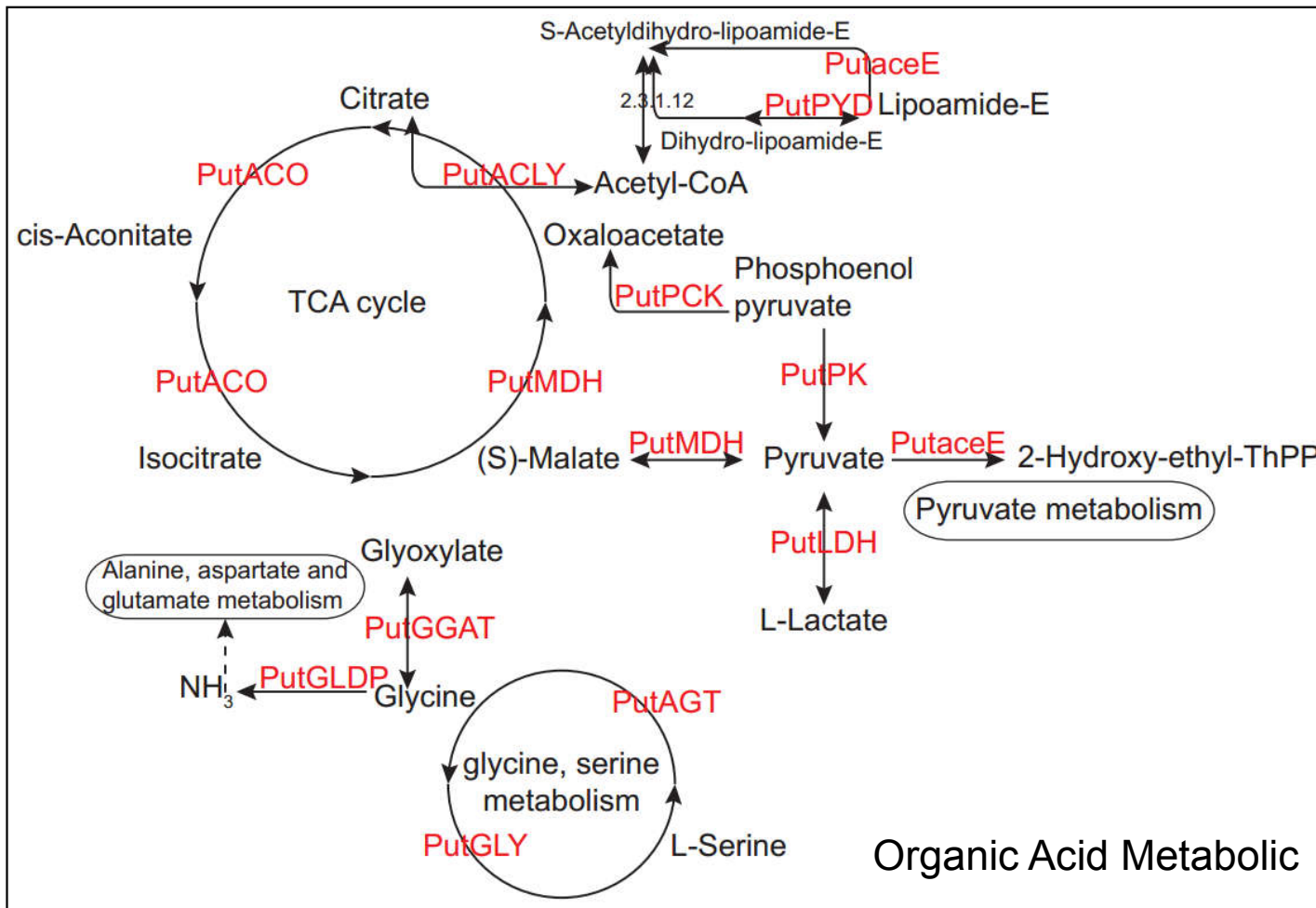

Supplement: Supplementary file 7 — Figure S7. Organic acid metabolism under saline-alkali conditions; the red font indicates the upregulated unigenes. (PDF 107 kb) [file 12864_2019_5860_MOESM7_ESM.pdf]

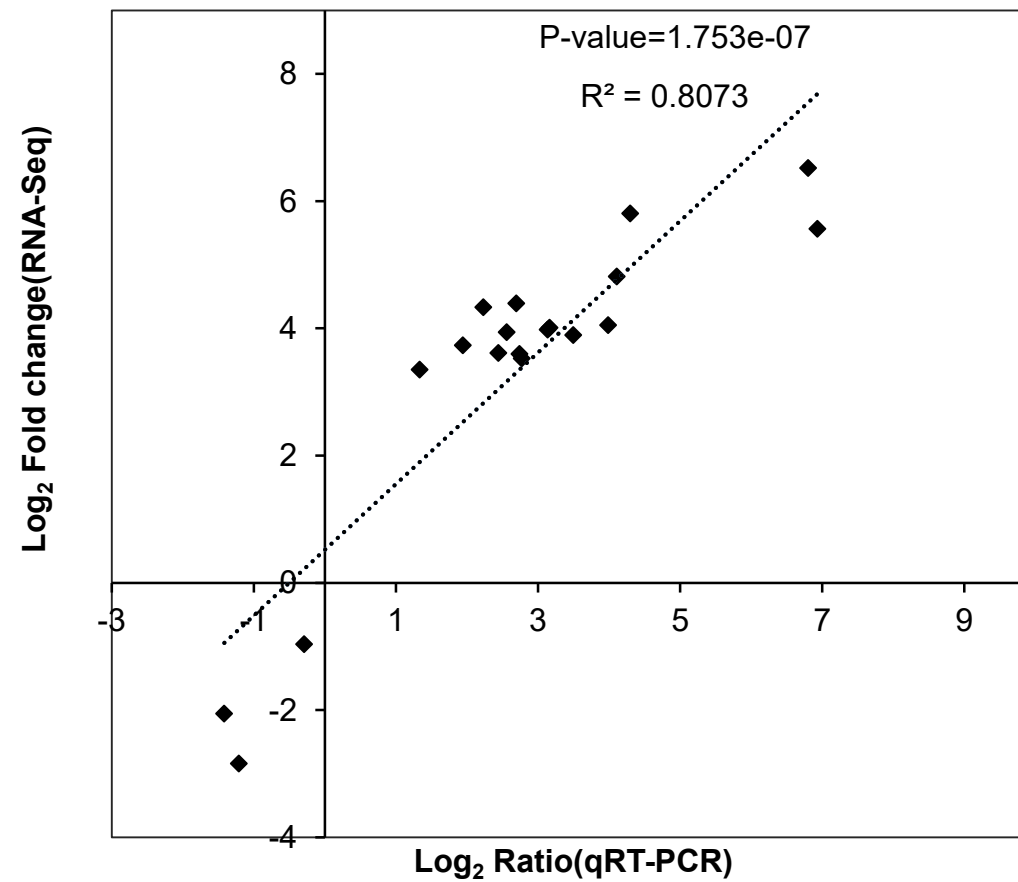

Supplement: Supplementary file 8 — Figure S8. Correlation analysis of 19 randomly selected DEGs based on the qRT-PCR and RNA-seq data. Pearson correlation coefficients are shown (R2 = 0.8073) (P < 0.001). (PDF 78 kb) [file 12864_2019_5860_MOESM8_ESM.pdf]
