# Supplementary material for: Transcriptome profiling of Puccinellia tenuiflora during seed germination under a long-term saline-alkali stress
Source: BMC Genomics. 2019 Jul 17;20:589. doi: 10.1186/s12864-019-5860-5 (PMC6637651; doi:10.1186/s12864-019-5860-5)
Supplement: Supplementary file 2 — Figure S2. BLAST results from the Nr database. (a) Similarity distribution. (b) Best-hit species classification. (c) E-value distribution. (PDF 120 kb) [file 12864_2019_5860_MOESM2_ESM.pdf]

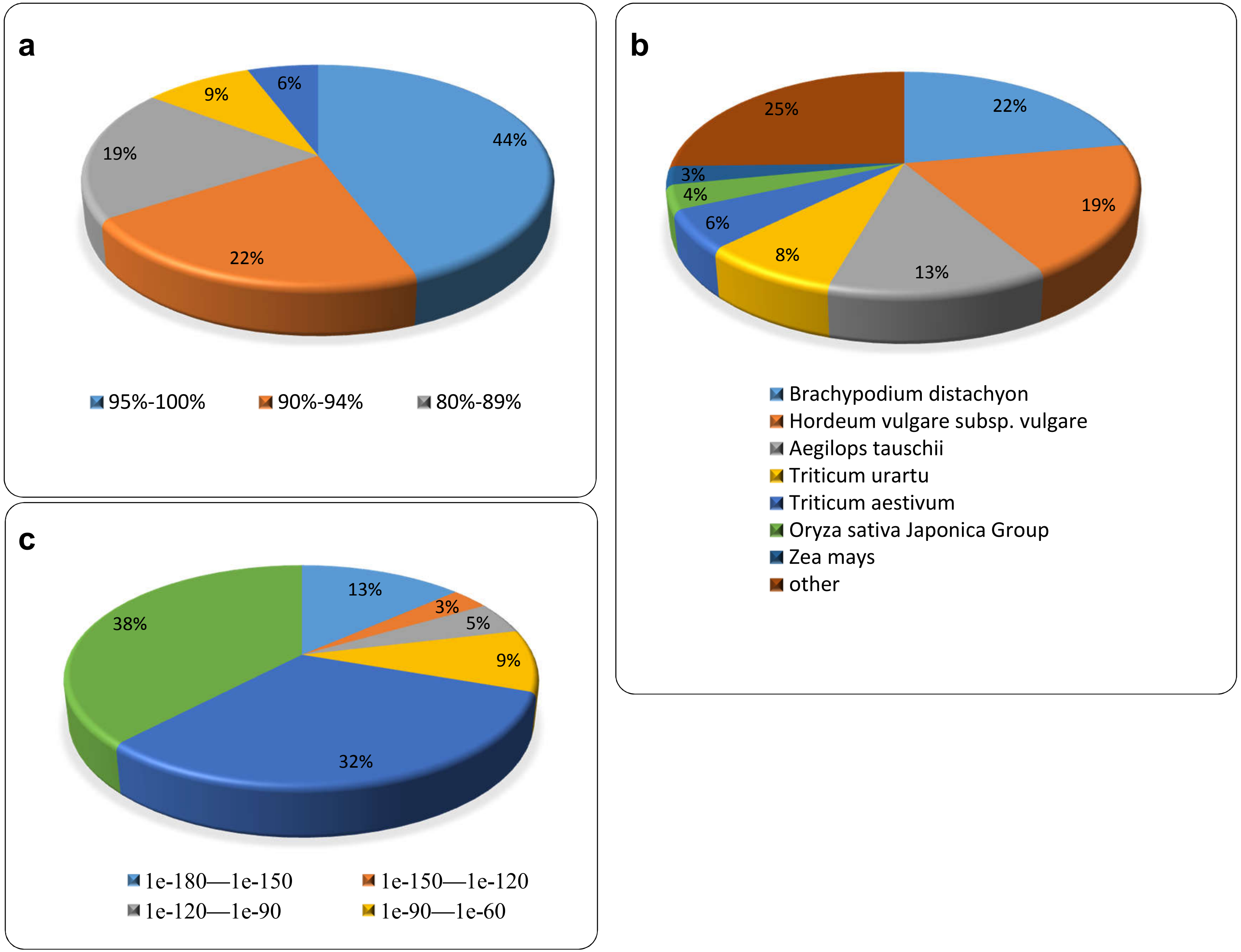
a
6%
9%
44%
19%
22%
95%-100%
90%-94%
80%-89%
b
25%
22%
3%
4%
6%
19%
8%
13%
Brachypodium distachyon
Hordeum vulgare subsp. vulgare
Aegilops tauschii
Triticum urartu
Triticum aestivum
Oryza sativa Japonica Group
Zea mays
other
c
13%
3%
38%
5%
9%
32%
1e-180—1e-150
1e-150—1e-120
1e-120—1e-90
1e-90—1e-60
