# Supplementary material for: Transcriptome profiling of Puccinellia tenuiflora during seed germination under a long-term saline-alkali stress
Source: BMC Genomics. 2019 Jul 17;20:589. doi: 10.1186/s12864-019-5860-5 (PMC6637651; doi:10.1186/s12864-019-5860-5)
Supplement: Supplementary file 4 — Figure S4. KEGG pathways of assembled unigenes from P. tenuiflora. The x-axis indicates the categories, and the y-axis indicates the number of unigenes. (PDF 189 kb) [file 12864_2019_5860_MOESM4_ESM.pdf]

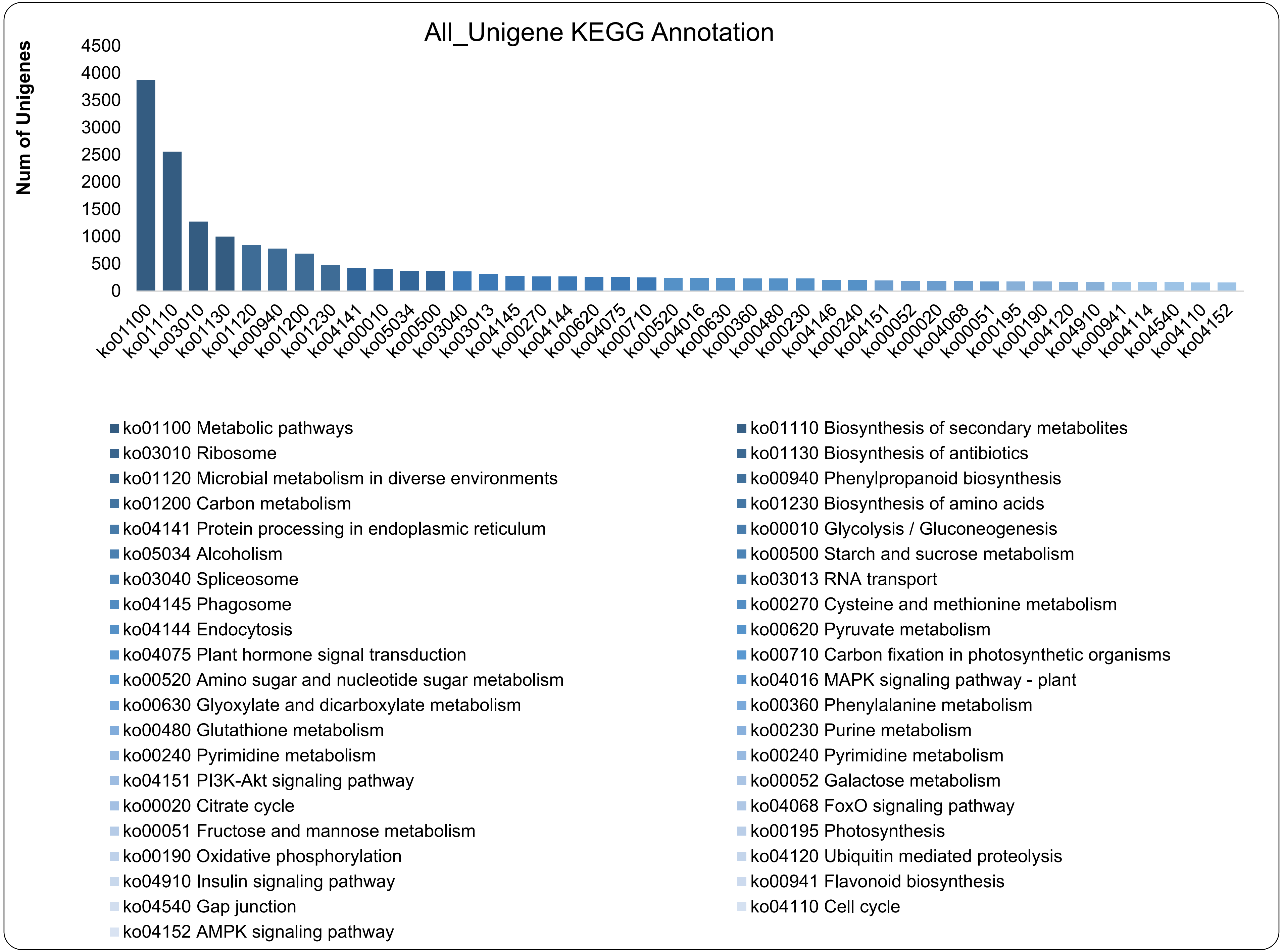
All_Unigene KEGG Annotation
Num of Unigenes
4500
4000
3500
3000
2500
2000
1500
1000
500
0
ko01100 ko01110 ko03010 ko01130 ko01120 ko00940 ko01200 ko01230 ko04141 ko00010 ko05034 ko00500 ko03040 ko03013 ko04145 ko00270 ko04144 ko00620 ko04075 ko00710 ko00520 ko04016 ko00630 ko00360 ko00480 ko00230 ko04146 ko00240 ko04151 ko00052 ko00020 ko04068 ko00051 ko00195 ko00190 ko04120 ko04910 ko00941 ko04114 ko04540 ko04110 ko04152
ko01100 Metabolic pathways
ko01110 Biosynthesis of secondary metabolites
ko03010 Ribosome
ko01130 Biosynthesis of antibiotics
ko01120 Microbial metabolism in diverse environments
ko00940 Phenylpropanoid biosynthesis
ko01200 Carbon metabolism
ko01230 Biosynthesis of amino acids
ko04141 Protein processing in endoplasmic reticulum
ko00010 Glycolysis / Gluconeogenesis
ko05034 Alcoholism
ko00500 Starch and sucrose metabolism
ko03040 Spliceosome
ko03013 RNA transport
ko04145 Phagosome
ko00270 Cysteine and methionine metabolism
ko04144 Endocytosis
ko00620 Pyruvate metabolism
ko04075 Plant hormone signal transduction
ko00710 Carbon fixation in photosynthetic organisms
ko00520 Amino sugar and nucleotide sugar metabolism
ko04016 MAPK signaling pathway - plant
ko00630 Glyoxylate and dicarboxylate metabolism
ko00360 Phenylalanine metabolism
ko00480 Glutathione metabolism
ko00230 Purine metabolism
ko00240 Pyrimidine metabolism
ko00240 Pyrimidine metabolism
ko04151 PI3K-Akt signaling pathway
ko00052 Galactose metabolism
ko00020 Citrate cycle
ko04068 FoxO signaling pathway
ko00051 Fructose and mannose metabolism
ko00195 Photosynthesis
ko00190 Oxidative phosphorylation
ko04120 Ubiquitin mediated proteolysis
ko04910 Insulin signaling pathway
ko00941 Flavonoid biosynthesis
ko04540 Gap junction
ko04110 Cell cycle
ko04152 AMPK signaling pathway
